# Isolated cortical vein thrombosis after nitrous oxide use in a young woman: a case report

**DOI:** 10.1186/s12883-020-01961-4

**Published:** 2020-10-20

**Authors:** Mao Liu, Jing Zhang, Bitao Bu

**Affiliations:** 1 Department of Neurology, Tongji Hospital, Tongji Medical College, Huazhong University of Science and Technology, Wuhan, P. R. China; 2 Department of Radiology, Tongji Hospital, Tongji Medical College, Huazhong University of Science and Technology, Wuhan, P. R. China

**Keywords:** Nitrous oxide, Subacute combined degeneration, Peripheral neuropathy, Isolated cortical vein thrombosis, Case report

## Abstract

**Background:**

Nitrous oxide has become a popular inhalant as abused substance by young Chinese people in recent years. It has been mainly associated with medical conditions including megaloblastic anemia and myeloneuropathy.

**Case presentation:**

We report a case of a 25-year-old high school graduate who had been abusing nitrous oxide for twenty months. She had a history of peripheral neuropathy and subacute combined degeneration in between. The young woman presented with headache, motor aphasia and right arm paralysis of eight hours after intermittently consuming nitrous oxide for one week. D-dimer was increased (1.1 mg/ml). Blood vitamin B12, folate, homocysteine and beta-HCG levels were normal. Head CT showed hemorrhagic infarction and subarachnoid hemorrhage. MR angiography and venography were normal. Head MRI identified left frontal isolated cortical vein thrombosis. Her muscle strength and verbal fluency significantly improved after initiation of Low Molecular Weight Heparin and serial head MRI showed continuous reduction in the size of thrombus.

**Conclusions:**

For the first time nitrous oxide use is found to be related to isolated cortical vein thrombosis. Public education regarding the potential consequences of abusing nitrous oxide especially in high-risk individuals is urgently needed.

## Background

Nitrous oxide has been widely used as an anesthetic agent in the medical field. However, previous studies have found its use is associated with various medical conditions including macrocytic anemia, peripheral neuropathy, subacute combined degeneration, deep vein thrombosis, pulmonary embolism, aortic thrombosis and cerebral venous sinus thrombosis [1–20]. Here for the first time we report a young woman with isolated cortical vein thrombosis related to nitrous oxide use.

## Case presentation

### Medical history

A 25-year-old Chinese woman presented with headache and difficulty in speaking and moving the right arm for the last eight hours. She used oral contraceptives once ten days ago and inhaled nitrous oxide outside with friends in the last week.

The patient began to use oxide intermittently twenty months ago. She gradually developed numbness and weakness of distal limbs, and balance difficulty, which significantly improved after vitamin B12 intake of one month. One year ago her numbness in the distal limbs subacutely deteriorated and ascended to both proximal thighs and the upper trunk. Medical record showed she had impaired superficial sensation of both hands, feet and the thorax region from T4 to T8 dermatome, impaired deep sensation of the lower limbs, and areflexia. MRI showed hyper-intensities involving the posterior and lateral column of the spine cord ranging from T5 to T11 vertebral body on T2 sequence (not shown). A diagnosis of peripheral neuropathy and subacute combined degeneration was made and her symptoms again improved significantly with vitamin B12 intake.

The patient did not use other substances and had no family history.

### Neurological examination

The patient had significant motor aphasia, mild right facial paralysis and right deviation of tongue. Muscle strength of the right upper limb is 0/5 with reduced tendon reflex compared with the left side. No apparent abnormalities in mental status, sensations and coordination were found.

### Diagnostic tests and treatment

Erythrocyte sedimentation rate (34 mm/H), c-reaction protein (21.1 mg/L), D-Dimer (1.14 ug/ml) and lactate dehydrogenase (314 U/L) were elevated. Complete blood count, liver enzyme, creatinine, urea nitrogen, troponin I, protein C, protein S, antinuclear antibodies, autoantibodies to extractable nuclear antigens, antiphospholipid antibodies, anti-neutrophil cytoplasmic antibodies, and beta-HCG were all within normal limits. Folate and vitamin B12 levels were normal. Homocysteine level was within the upper limit (13.6 umol/L; normal range 6–14 umol/L). No mutation of Factor V Leiden was found. Methylenetetrahydrofolate reductase (MTHFR) genotype of C677T and A1298C, and plasminogen activator inhibitor 1 (PAI-1) genotype of 4G/5G were confirmed.

**Fig. 1:**
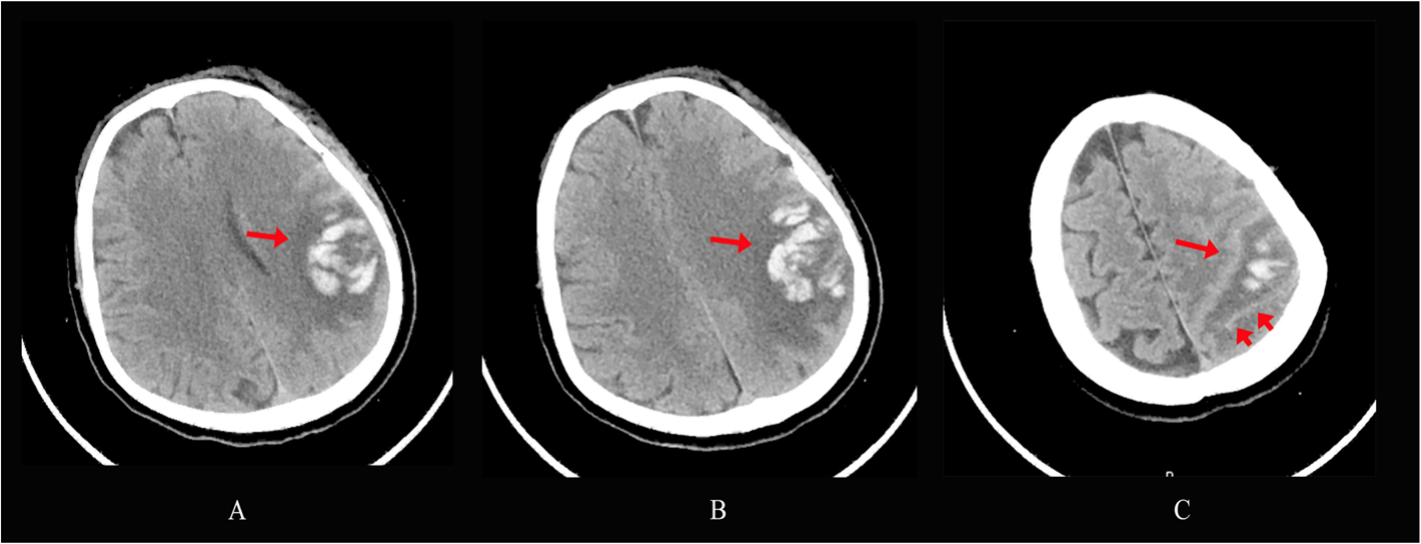
Head CT upon admission showed hemorrhagic infarction mainly involving the left parietal lobe (**a**-**c**, arrow) and subarachnoid hemorrhage (**c**, arrow heads)

**Fig. 2:**
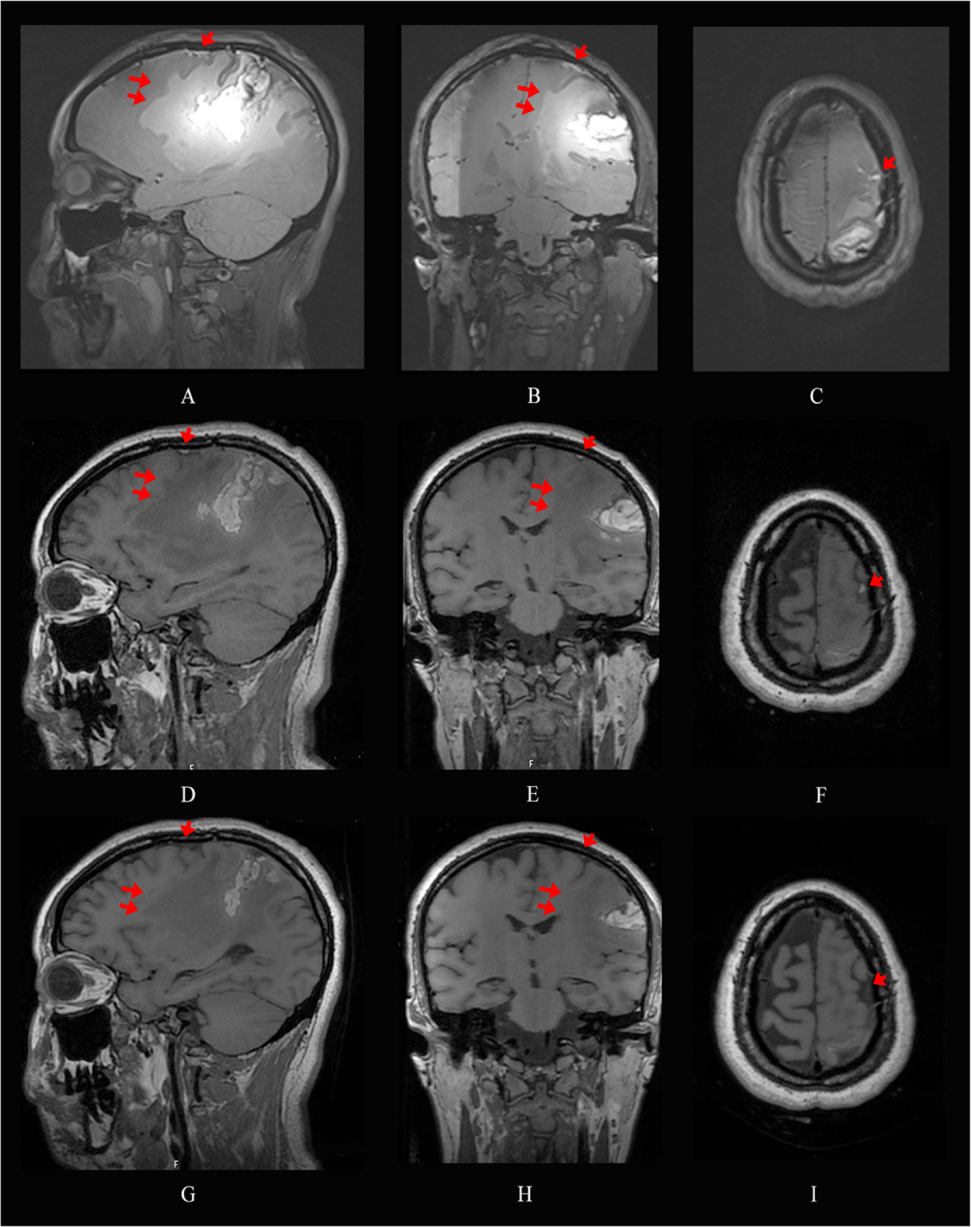
Head MRI ten days after admission showed isolated cortical vein thrombosis (arrow head) over the frontal cortical surface and expanding hematoma and edema (double arrow head) that caused cingulate herniation in the sagittal, coronal and axial section (**a**-**c**) on T2 SPACE sequence. The sizes of thrombosis (arrow head), hematoma and edema (double arrow head) continuously decreased on T1 CUBE sequence after five (**d**-**f**) and twelve (**g**-**i**) days of anticoagulation, respectively

Head CT showed hemorrhagic infarction (Fig. 1a&b, arrow) and subarachnoid hemorrhage (Fig. 1b, arrow heads) mainly involving the left parietal lobe. MR angiography and venography were normal (not shown) and excluded major abnormalities of the large and medium arteries as well as the venous sinus system. Since the patient did not experience any major symptomatic improvement during the hospital stay and there was the concern about the existence of isolated cortical vein thrombosis, MRI head with 0.1 mm thickness demonstrated expanding hematoma and edema causing cingulate herniation, and confirmed isolated cortical vein thrombosis over the frontal cortical surface in the sagittal, coronal and axial section (Fig. 2a-c) ten days after admission.

Vitamin B6, B12, folate, mannitol and Low Molecular Weight Heparin were initiated. The sizes of thrombus, hematoma and edema reduced continuously after treatment of five (Fig. 2d-f) and twelve days (Fig. 2g-i), respectively, and the patient achieved significant improvement in muscle strength (grade 3/5) and verbal fluency in the meantime. Anticoagulation was switched from Low Molecular Weight Heparin to oral anticoagulant after another two weeks and the patient was discharged.

### Outcome

During the last follow-up (6 weeks after symptom onset), the patient had further improvement in muscle strength (grade 4/5) and almost normalized verbal fluency. The patient was satisfied with her recovery and no adverse effects were reported.

## Discussion and conclusion

In this report we described a young Chinese woman with isolated cortical vein thrombosis [21] related to nitrous oxide use. Her history of peripheral neuropathy and subacute combined degeneration was most probably also attributed to nitrous oxide. To our knowledge this is the first case report on the association between nitrous oxide use and isolated cortical vein thrombosis in the existing literatures.

Nitrous oxide has gained its popularity among young people worldwide for many years [22] and also among Chinese people in recent years. Nitrous oxide produces analgesic and anxiolytic effects upon inhalation [22] and has abuse potential [13]. Nitrous oxide could potently change cobalamin from reduced to oxidized state [23]; the lack of bioavailable cobalamin disrupts the normal function of methionine synthase which requires both reduced cobalamin and folate as cofactors [24]. Reduced methionine further leads to reduced S-adenosylmethionine production, which is associated with defective DNA maturation, megaloblastic changes of red blood cells [24] and abnormal myelination of nerve fibers [25]. This is supported by numerous previous individual reports and systematic reviews on the incidence of megaloblastic anemia, peripheral neuropathy and subacute combined degeneration related to nitrous oxide [1–16], and also in line with a history of peripheral neuropathy and subacute combined degeneration of our patient.

Much less frequently reported were incidences of thrombotic events related to chronic use of nitrous oxide. Den Uil et al. reported a 32-year-old male with aortic arch thrombus formation, increased homocysteine and decreased vitamin B level, and a heterozygous factor II mutation [19]. Bajaj et al. described a 32-year-old male with ischemic stroke, decreased vitamin B12 and folate level, and increased homocysteine level [20]. Sun et al. reported a 29-year-old male with deep vein thrombosis, pulmonary embolism and homocysteinemia [18]. Pratt et al. presented a 21-year-old female with a large cerebral venous sinus thrombosis with elevated homocysteine and methylmalonic acid level, normal cobalamin and folate levels. The patient was also pregnant and had significant MTHFR polymorphisms [17]. Previous studies showed that MTHFR genotype polymorphisms including C677T and A1298C were associated with elevated homocysteine levels [26, 27]; homocysteinemia was again associated with increased risk of venous thrombosis [28, 29]. Furthermore, oral contraceptive has been associated with increased thrombotic risk in women [30, 31]. While in all the above mentioned case reports there was consistent elevation of homocysteine level, our patient only had a high normal homocysteine level. Our patient had normal protein C, protein S and beta-HCG levels; Factor V Leiden mutation was not found. Thus, it cannot be excluded that the use of nitrous oxide by our patient in the preceding week of stroke still increased the homocysteine level (homocysteine level was 9 umol/L one year ago when the diagnosis of subacute combined degeneration was made) and contributed to a hypercoagulable state [32] together with oral contraceptive use. Our patient also had PAI-1 4G/5G polymorphism. However, it is to note that a recent study did not find a higher risk of venous thrombosis related to PAI-1 4G/5G polymorphism [33], and it is uncertain what role it played in the thrombotic event in our patient.

A major limitation is that we could not screen for all types of hereditary thrombophilia to exclude effects of other unknown conditions. However, considering the close temporal relationship between nitrous oxide use and the incidence of isolated cortical vein thrombosis in our patient who had a clear past history of medical conditions associated with nitrous oxide use, we believe nitrous oxide use had at least partly contributed to the formation of isolated cortical vein thrombosis in our patient despite of scare reports from existing literatures [17–20].

In conclusion, nitrous oxide use could be associated with thrombotic events including isolated cortical vein thrombosis, especially in individuals with other factors predisposing to a hypercoagulable state apart from diseases of the hematological and neurological systems. Early recognition and treatment of isolated cortical vein thrombosis is critical. Considering its easy accessibility among young people, it is of great importance to raise public consciousness of the potential negative effects of nitrous oxide use on young individuals.

## Data Availability

Data sharing is not applicable to this article as no datasets were generated or analyzed for this study.

## Abbreviations

MTHFR: Methylenetetrahydrofolate reductase
PAI-1: Plasminogen activator inhibitor 1
